# Treatment with Cannabinoids as a Promising Approach for Impairing Fibroblast Activation and Prostate Cancer Progression

**DOI:** 10.3390/ijms21030787

**Published:** 2020-01-25

**Authors:** Laura Pietrovito, Marta Iozzo, Marina Bacci, Elisa Giannoni, Paola Chiarugi

**Affiliations:** Department of Experimental and Clinical Biomedical Sciences, University of Florence, 50134 Florence, Italy; marta.iozzo@student.unisi.it (M.I.); marina.bacci@unifi.it (M.B.); paola.chiarugi@unifi.it (P.C.)

**Keywords:** WIN 55-212.2 mesylate, cannabidiol, endocannabinoids, cancer-associated fibroblasts, prostate cancer

## Abstract

Endo-, phyto- and synthetic cannabinoids have been proposed as promising anti-cancer agents able to impair cancer cells’ behavior without affecting their non-transformed counterparts. However, cancer outcome depends not only on cancer cells’ activity, but also on the stromal cells, which coevolve with cancer cells to sustain tumor progression. Here, we show for the first time that cannabinoid treatment impairs the activation and the reactivity of cancer-associated fibroblasts (CAFs), the most represented stromal component of prostate tumor microenvironment. Using prostate cancer-derived CAFs, we demonstrated that WIN 55-212.2 mesylate, a synthetic full agonist of cannabinoid receptors (CBs) 1 and 2, downregulates α-smooth muscle actin and matrix metalloprotease-2 expression, and it inhibits CAF migration, essential features to ensure the activated and reactive CAF phenotype. Furthermore, by impairing stromal reactivity, WIN 55-212.2 mesylate also negatively affects CAF-mediated cancer cells’ invasiveness. Using selective antagonists of CBs, we proved that CAFs response to WIN 55-212.2 mesylate is mainly mediated by CB_2_. Finally, we suggest that endocannabinoids self-sustain both prostate tumor cells migration and CAFs phenotype by an autocrine loop. Overall, our data strongly support the use of cannabinoids as anti-tumor agents in prostate cancer, since they are able to simultaneously strike both cancer and stromal cells.

## 1. Introduction

Cannabinoids are bioactive lipids able to interact with specific cell-surface cannabinoid receptors (CBs). They can be divided into three main classes: endocannabinoids (endogenous ligands), phytocannabinoids (derived from *Cannabis Sativa*), and synthetic cannabinoids. In the last few decades, growing evidence has reported their ability to impair cancer progression both in vitro and in xenograft models of human cancers. The anti-tumorigenic activities include: (i) inhibition of cancer cells’ proliferation and migration, (ii) induction of cancer cell death, (iii) impairment of neoangiogenesis, and (iv) modulation of anti-tumor immune response [1,2,3]. Δ-9-tetrahydrocannabinol (THC) and cannabidiol (CBD) are the two best-characterized active components contained in marijuana. THC binds with high affinity both CB_1_ and CB_2_, while CBD, which lacks psychotropic activity, mainly interacts with vanilloid receptor 1 (TRPV1). Of interest, CB_1_ and CB_2_ were found up-regulated in malignant tissues compared to their non-transformed counterparts and a correlation between high expression and poor prognosis has been proven for different human tumors [4]. 

Cannabinoids have been shown to be promising anticancer agents in prostate cancer treatment [5]. In prostate cancer patients, high expression of CB_1_ has been associated with a worse prognosis [6,7], and several authors reported CB_1_-mediated anti-proliferative and anti-invasive effects of cannabinoids in prostate cancer cells [8,9,10,11,12]. Furthermore, it has been recently demonstrated that CB_2_ also controls tumor cells proliferation, migration, and invasion in both prostate cancer cell lines and in vivo models [13]. 

To date, it is widely accepted that cancer outcome does not only depend on the behavior of cancer cells, but also on the tumor microenvironment (TME) that coevolves with cancer cells, sustaining the enhancement of tumor malignancy. We previously demonstrated that cancer-associated fibroblasts (CAFs), the most represented stromal cells in the prostate TME, play an intriguing role during all stages of disease progression, including metastasis [14,15,16,17,18,19,20]. Herein, we show a selective action of WIN 55-212.2 mesylate, a synthetic cannabinoid with affinity for CB_1_ and CB_2_ higher than THC, on prostate cancer cell lines without affecting healthy counterpart. Furthermore, we demonstrated for the first time that patient-derived prostate CAFs upregulate CBs expression compared to normal fibroblasts (HPFs). WIN 55-212.2 mesylate strongly impaired CAFs’ activation and CAFs-induced tumor invasion. By using selective antagonists of CB_1_ and CB_2_, we proved that WIN 55-212.2 mesylate operated mainly through CB_2_. Finally, we demonstrated that migration of androgen-independent prostate cancer cells (PC-3 and DU-145) and CAFs phenotype could be regulated by an autocrine self-sustaining loop of endocannabinoids. 

Overall, these data support the use of cannabinoids as promising anti-cancer drugs in prostate cancer patients, since they are able to simultaneously strike cancer and stromal cells. 

## 2. Results

### 2.1. WIN 55-212.2 Mesylate Selectively Impairs Prostate Cancer Cells Survival

**Figure 1:**
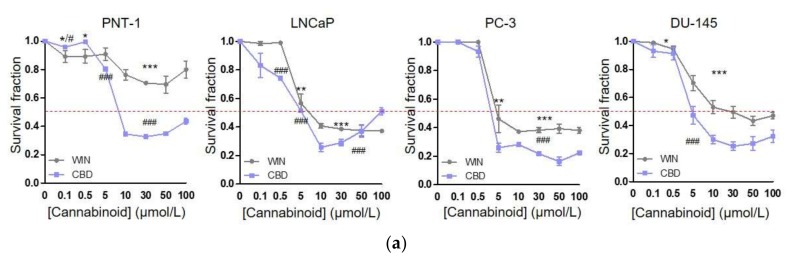

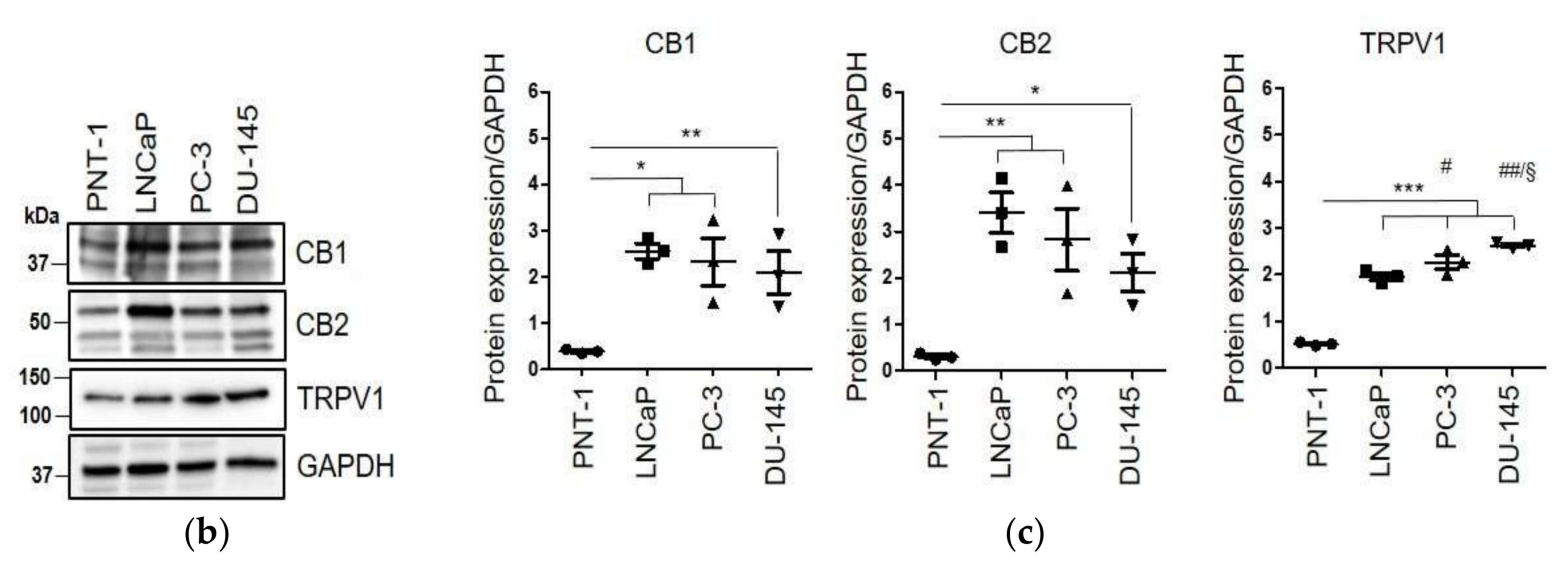
WIN 55-212.2 mesylate impacts on prostate cancer cell survival without affecting healthy prostate epithelial cells (**a**) Normal prostate epithelial cells (PNT-1), androgen-sensitive (LNCaP), and insensitive (PC-3 and DU-145) prostate cancer cells were incubated with increasing concentrations of WIN 55-22.2 mesylate and CBD for 24 h. Cell survival measurements by means of data obtained by crystal violet staining are shown (*n* = 3). *p*-Values were determined by Student’s *t* test. * *p* < 0.05, ** *p* < 0.01, *** *p* < 0.001 WIN versus control (Untr); # *p* < 0.05, ### *p* < 0.001 CBD versus Untr. (**b**) Representative immunoblot of CB_1_, CB_2_ and TRPV1 expressions in PNT-1 and prostate cancer cell lines (**c**) Quantitative results of western blot analysis. Relative expressions were normalized respect to GAPDH. Each dot represents a biological replicate. Data express means ± SEMs. *p*-values were calculated by one-way ANOVA followed by Bonferroni’s multiple comparison test. * *p* < 0.05, ** *p* < 0.01, *** *p* < 0.001 versus PNT-1; # *p* < 0.05, ## *p* < 0.01 versus LNCaP; § *p* < 0.05 versus PC-3.

The best-characterized anti-tumoral activities of cannabinoids are their ability to induce cancer cell death by apoptosis and to inhibit cancer cell proliferation [3,21,22]. In keeping with those, we evaluated the anti-proliferative effects of WIN 55-212.2 mesylate and CBD in both androgen-sensitive (LNCaP) and insensitive (PC-3 and DU-145) prostate cancer cells. As a control, we used healthy prostate epithelial cells (PNT-1). Tumor and control cells were treated for 24 h with concentrations of WIN 55-212.2 mesylate and CBD ranging from 0.5 to 100 µM, and then cell viability was analyzed by crystal violet staining (Figure 1a). Our results show that WIN 55-212.2 mesylate at concentrations ≥ 5 µM is a selective anti-tumor compound, able to kill tumor cells without affecting their non-transformed counterparts. Interestingly, CBD 5 µM reduces almost 50% of tumor cells’ viability, while PNT-1 survival decreases only 20%. Then, we evaluated CBs’ and TRPV1’s expression in our cell models (Figure 1b). Western blot analysis revealed that the normal prostate epithelial cells express similar levels of CB_1_ and TRPV1, while exhibiting lower levels of CB_2_. All cannabinoid receptors strongly increase in tumor cells with respect to PNT-1. Interestingly, CB_1_ expression and CB_2_ expression are both higher in LNCaP compared to PC-3 and DU-145 cells, while TRPV1 is modulated in an opposite manner. 

### 2.2. CAFs Derived from Aggressive Prostate Cancer Bearing Patients Upregulate CB_1_ and CB_2_ Compared to Normal Fibroblasts

**Figure 2:**
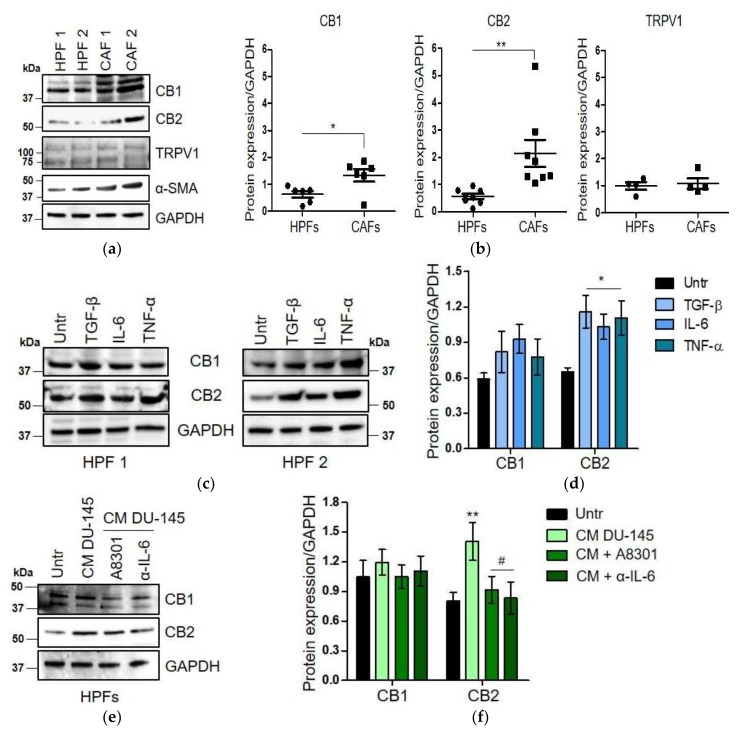
Prostate cancer patient-derived CAFs upregulate CB_1_ and CB_2_ receptors in response to inflammatory cytokines. (**a**) Representative immunoblot of CBs, TRPV1, and α-SMA expression in normal fibroblasts (HPFs) and cancer-associated fibroblasts (CAFs) isolated from two different explants (HPF1/HPF2; CAF1/CAF2). (**b**) Quantitative results of western blot analysis performed in HPFs (*n* = 3) and CAFS (*n* = 4). Relative expressions were normalized respect to GAPDH. Each dot represents a biological replicate (*n* = 6 for CB_1_, *n* = 7 for CB_2_, *n* = 4 for TRPV1). Data represent means ± SEMs. *p*-values were calculated by Student’s *t* test. * *p* < 0.05, ** *p* < 0.01 versus HPFs. (**c**) Expression of CBs in two different primary HPFs (HPF1/HPF2) stimulated for 48 h with TGF-β 10 ng/mL, IL-6 100 ng/mL, and TNF-α 25 ng/mL. Cells maintained in serum-deprived medium have been used as control (Untr). (**d**) Quantitative results of western blot analysis performed twice on two different patient-derived HPFs. Data represent means ± SEMs. One-way ANOVA, Dunnet’s corrected. * *p* < 0.05 versus Untr. (**e**) Representative immunoblot of CBs expression on HPFs treated for 48 h with the conditioned media (CM) from DU-145 cells, in presence or not of TGF-β receptor inhibitor (A8301, 1 µM) and neutralizing antibodies for IL-6 (α-IL-6, 5 µg/mL). Cells were pre-treated with inhibitors for 1 h, and then the CM from tumor cells was added. Cells maintained in serum-deprived medium have been used as control (Untr). (**f**) Quantitative results of western blot analysis performed twice on HPFs obtained from two different explants. Data represent means ± SEMs. One-way ANOVA, Dunnet’s corrected. ** *p* < 0.01 versus Untr; # *p* < 0.05 versus CM-DU-145.

The expression of CBs and TRPV1 has been evaluated in primary patient-derived fibroblasts. CAFs were isolated from aggressive prostate cancer bearing patients (Gleason score 4 + 5, 4 + 4, grade ≤ pT3), while normal fibroblasts were obtained from the adjacent non-neoplastic area. Western blot analysis indicates that CB_1_ and CB_2_ are up-regulated in CAFs compared to HPFs, while TRPV1 is weakly expressed in both cell types, without significant differences between them (Figure 2a,b). To investigate the regulation of CBs during activation of normal fibroblasts in the CAFs phenotype, HPFs were stimulated for 48 h with three different inflammatory cytokines: transforming growth factor (TGF)-β, interleukin (IL)-6, and tumor necrosis factor (TNF)-α. Indeed, prostate cancer cells release high concentrations of TGF-β and IL-6 inside the TME, which play a mandatory role in the activation of resident fibroblasts into CAFs [14,23,24,25]. Concerning TNF-α, it has been recently proven that it is able to modulate the CB_2_ expression in human T cells and PBMCs [26]. Our results show that all the tested cytokines are able to induce CBs expression, with significant increase in CB_2_ levels (Figure 2c,d). In keeping with the high abundance of IL-6 and TGF-β in prostate cancer cells’ secretome, conditioned media from DU-145 cells also upregulate both CB_1_ and CB_2_ expression in treated HPFs (Figure 2e). To confirm the role of tumor-secreted IL-6 and TGF-β in modulating CBs expression, HPFs were treated with conditioned media from DU-145 cells in the presence of and without blocking antibodies for IL-6 (α-IL-6) and a selective inhibitor of TGF-β receptor 1 (A8301). We previously proved that α-IL-6 is functional at the concentration of 5 µg/mL [27], while the dose 1 µM of A8301 was established by evaluating activation of SMAD2/3 in a dose-response experiment (Figure S1a). As expected, CB_2_ expression is regulated by both IL-6 and TGF-β pathways (Figure 2e). 

### 2.3. WIN 55-212.2 Mesylate Impacts on CAFs Phenotype and Impairs TGF-β-Induced Activation in Normal Fibroblasts via CB_2_ Receptor

**Figure 3:**
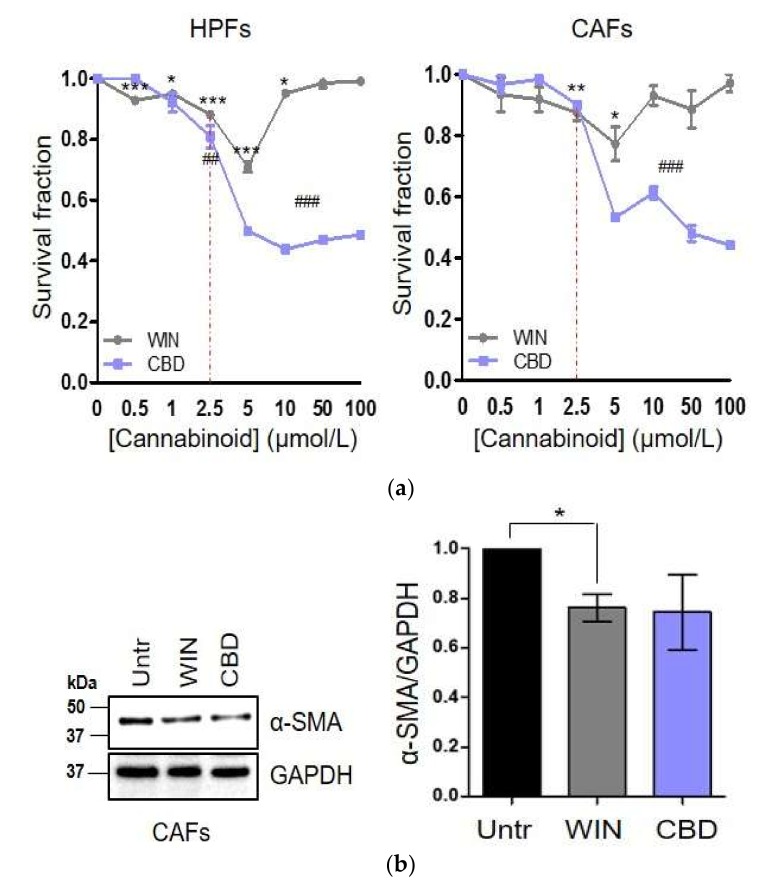

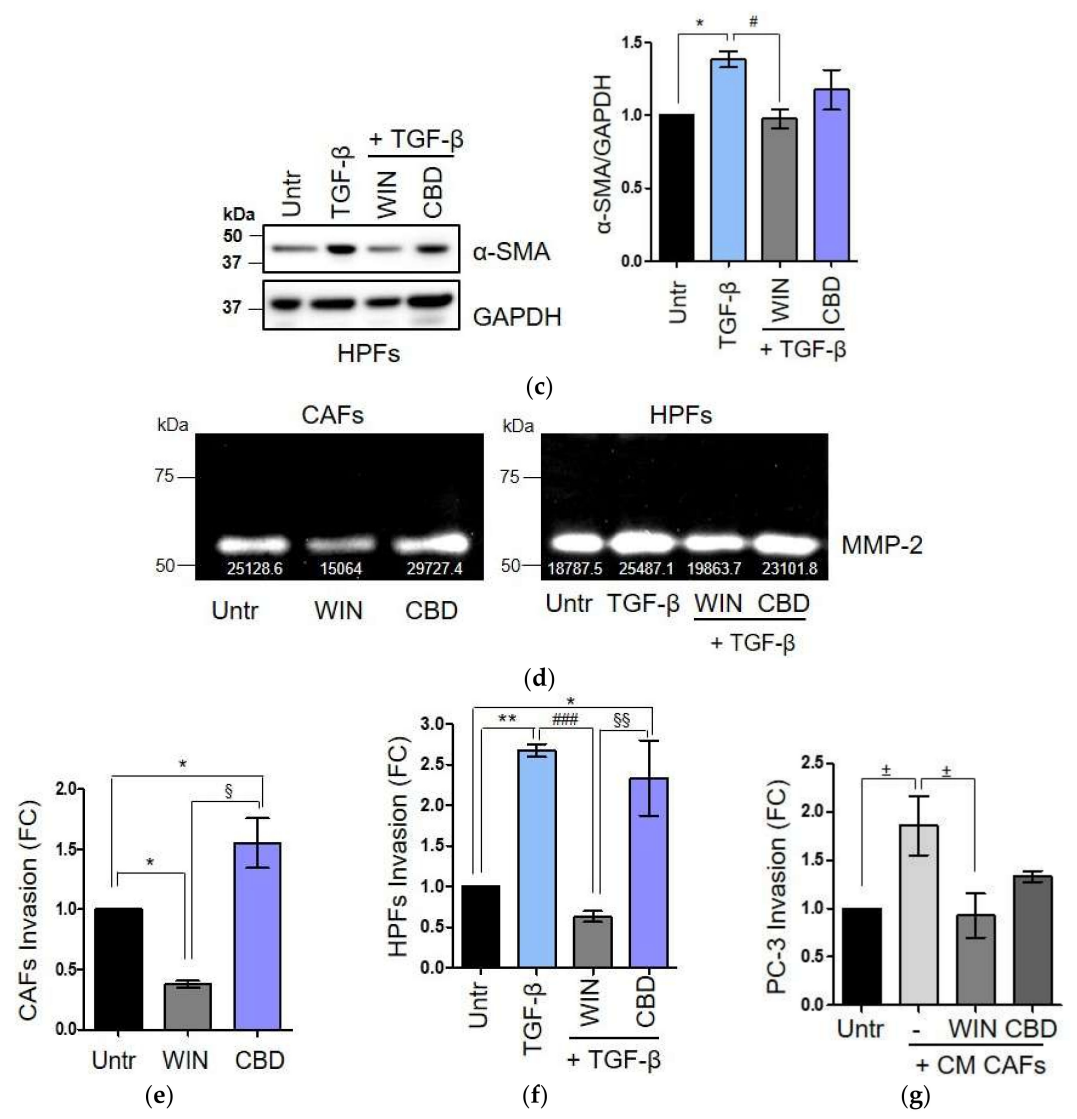
WIN 55-212.2 mesylate affects CAFs’ reactivity and impairs TGF-β-mediated activation of HPFs. (**a**) HPFs and CAFs were treated with increasing concentrations of WIN 55-212.2 mesylate and CBD for 24 h; then, cells survival was determined by crystal violet staining. Data represent means ± SEMs of three independent biological replicates. p-values were determined by Student’s *t* test. * *p* < 0.05, ** *p* < 0.01, *** *p* < 0.001 WIN versus control (Untr); ## *p* < 0.01, ### *p* < 0.001 CBD versus Untr. We choose cannabinoid concentration at 2.5 µM to perform all functional assay. (**b**,**c**) Western blot analysis and quantitative measurement of α-SMA expression in CAFs (upper) and HPFs activated in vitro by TGF-β 10 ng/mL (lower) treated with WIN 55-212.2 mesylate and CBD 2.5 µM for 24 h. Data represent means ± SEMs of three independent biological replicates performed on fibroblasts isolated from two different explants. Relative α-SMA expressions were normalized respect to GAPDH and represented as FC versus control Untr. p-values were calculated by one-way ANOVA followed by Bonferroni’s multiple comparison test. * *p* < 0.05 versus Untr; # *p* < 0.05 versus TGF-β. (**d**) Gelatin zymography of conditioned media (CM) 10 fold concentrated from CAFs (left) and HPFs (right) treated like above. The white numbers in bolt indicate quantification of two independent biological replicates. (**e**,**f**) 1 × 10^5^ CAFs (left) or HPFs activated in vitro by TGF-β 10 ng/mL (right) were treated with cannabinoids 2.5 µM and allowed to invade toward complete medium (10% FBS) for 24 h. Data represent means ± SEMs of three biological replicates. One-way ANOVA, Bonferroni’s corrected. * *p* < 0.05, ** *p* < 0.01 versus Untr; § *p* < 0.05, §§ *p* < 0.01 versus WIN; ### *p* < 0.001 versus TGF-β. (**g**) 3 × 10^4^ PC-3 cells were incubated with CM from CAFs treated or not with cannabinoids 2.5 µM and allowed to invade toward complete medium. Starved cells have been used as negative control (Untr). Results express means ± SEMs of three independent biological replicates performed with CM from two different CAFs. One-way ANOVA, Dunnet’s corrected. ± *p* < 0.05 versus CM CAFs.

In line with differential expression of CBs between HPFs and CAFs, we tested if the treatment with WIN 55-212.2 mesylate and CBD could differentially affect HPFs and CAFs survival. We assessed the survival rate following 24 h of treatment with cannabinoids, with concentrations ranging from 0.5 µM to 100 µM (Figure 3a). Surprisingly, despite the weak expression of TRPV1, both HPFs and CAFs are more sensitive to CBD than WIN 55-212.2 mesylate treatment. This effect could be due to the expression of other receptors for which CBD binds as a full agonist, such as peroxisome proliferator-activated receptor (PPAR)-γ and serotonin receptor 5-HT [28]. In contrast, HPFs and CAFs viability is similarly and poorly affected by WIN 55-212.2 mesylate, underling that CBs do not likely regulate survival pathways in these stromal cells. 

Therefore, we evaluated if cannabinoids treatment could impair CAFs activation and reactivity. In according to the literature, we choose cannabinoid concentration at 2.5 µM to perform functional assay, since this concentration does not affect cell survival of both cancer cells and fibroblasts. CAFs, as well as HPFs activated in vitro with TGF-β, were treated with WIN 55-212.2 mesylate and CBD 2.5 µM for 24 h and then analyzed by a panel of functional assays. Our results show that the treatment of CAFs with WIN 55-212.2 mesylate reduces α-smooth muscle actin (SMA) and matrix metalloproteinase (MMP)-2 expression (Figure 3b,d) and decreased CAFs invasion abilities (Figure 3e). Similarly, in TGF-β-activated HPFs, WIN 55-212.2 mesylate 2.5 µM is able to impair TGF-β-activation, preventing the induction of α-SMA and MMP-2 expression (Figure 3c,d) and invasion abilities (Figure 3f). 

We previously demonstrated that CAFs reactivity is crucial to induce an epithelial to mesenchymal transition (EMT) of PC-3 cells gaining them with pro-invasive capabilities [14,29,30]. Thus, to evaluate if cannabinoids could affect also the pro-invasive activity of CAFs, PC-3 cells were exposed for 48 h to the conditioned media obtained from CAFs treated or not with cannabinoids. Our results demonstrate that WIN 55-212.2 mesylate severely reduces CAFs-induced invasiveness of PC-3 cells (Figure 3g). The inhibition of tumor cell invasion triggers by CBD is probably due to high expression of TRPV1 in androgen-insensitive cells. 

**Figure 4:**
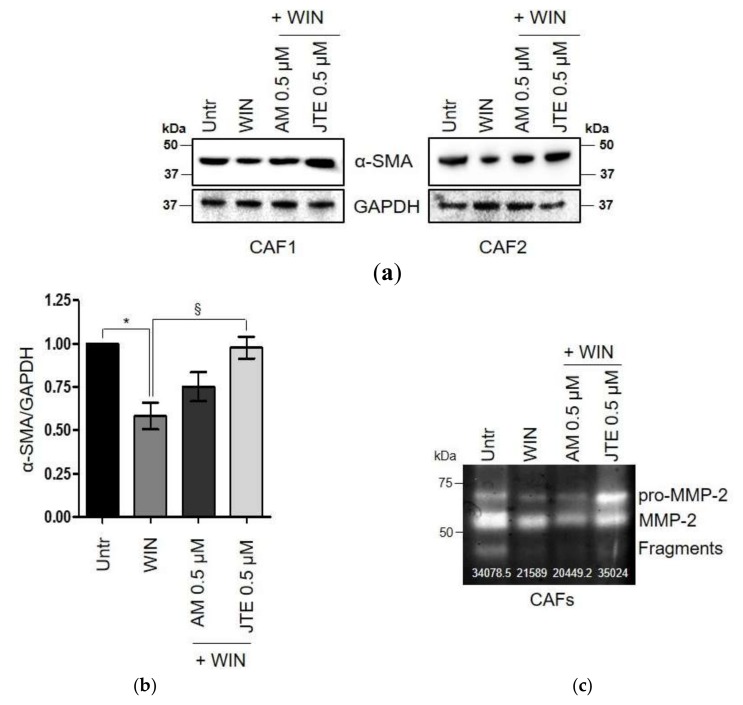
The effect of WIN 55-212.2 mesylate on CAFs phenotype is mainly mediated by CB_2_. (**a**) Representative immunoblot of α-SMA expression in CAFs isolated from two different explants (CAF1/CAF2) treated with WIN 55-212.2 mesylate 2.5 µM for 24 h in presence or not of AM281 and JTE-907 0.5 µM. Cells were pre-treated for 3 h with the antagonists, then WIN 55-212.2 mesylate was added. (**b**) Quantitative results of western blot analysis. Relative expressions were normalized with respect to GAPDH and shown as FC respect to control cells maintained in serum-deprived medium (Untr). Data represent means ± SEMs of three independent biological replicates performed on two different patient-derived CAFs. One-way ANOVA, Bonferroni’ corrected. * *p* < 0.05 versus Untr; § *p* < 0.05 versus WIN. (**c**) Gelatin zymography of conditioned media (10 fold concentrated) from CAFs treated as above. The white numbers in bolt indicate quantification of two independent biological replicates.

Finally, to address which CB modulates the effects of WIN 55-212.2 mesylate in ex vivo cultures of human CAFs, fibroblasts were pre-treated with selective antagonists of CB_1_ and CB_2_, AM281 and JTE-907 respectively, and analyzed for changes in α-SMA and MMP-2 expression. We chose the antagonists concentration to be 0.5 µM by evaluating ERK1/2 phosphorylation in a dose-response experiment (Figure S1b). Cell viability following 24 h of incubation with CBs antagonists was also assessed (Figure S1c). Pre-treatment with JTE-907, followed by the treatment with WIN 55-212.2 mesylate prevented the downregulation of both the proteins induced by the synthetic cannabinoid, underlining an involvement of CB_2_ receptors in mediating CAFs response to WIN 55-212.2 mesylate Figure 4a,b).

### 2.4. The Endocannabinoids Self-Sustain the Migration of Androgen-Insensitive Prostate Cancer Cells and CAFs’ Reactivity by an Autocrine Loop

**Figure 5:**
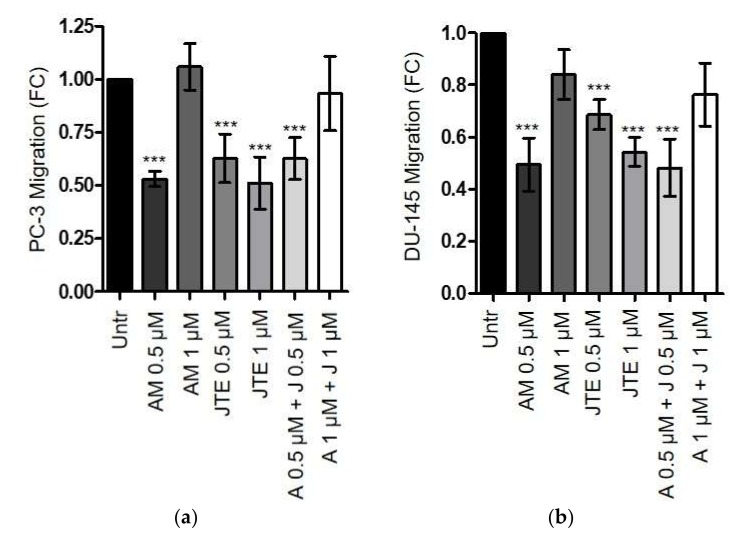

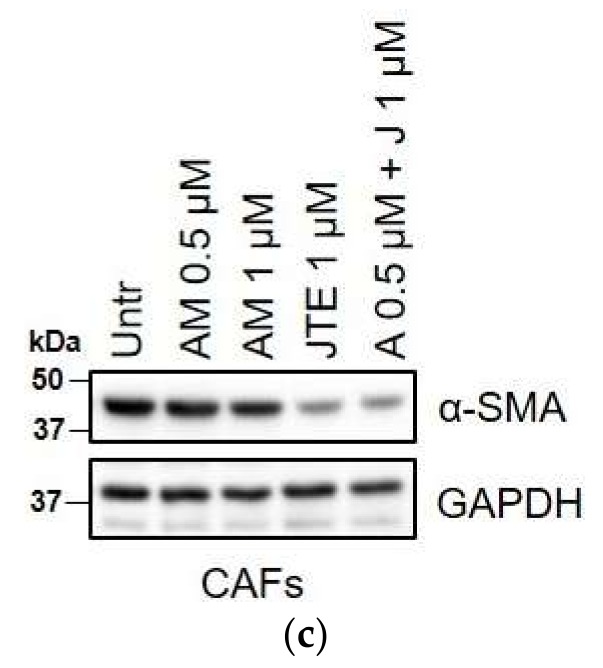
The endocannabinoids affect the migration abilities of androgen-insensitive prostate cancer cells and CAFs’ reactivity by autocrine self-sustaining loops. (**a**,**b**) 3 × 10^4^ PC-3 (left) or DU-145 (right) cells were pre-treated for 24 h with different concentrations of AM281 and JTE-907 administrated alone or in combination. Tumor cells were allowed to migrate for 16 h towards complete medium (10% FBS). Cells maintained in serum-deprived medium were used as controls (Untr). Data represent means ± SEMs of three independent biological replicates. *** *p* < 0.001 versus Untr. (**c**) Representative immunoblot of α-SMA expression on CAFs treated with different concentrations of CBs antagonists, administrated alone or in combination, for 24 h.

Several studies have already described the anti-proliferative effects of exogenous administrated endocannabinoids in prostate cancer cells [6,7,8,9,10,11,12]. The two primary endocannabinoids are *N*-arachidonoylethanolamine (anandamide, AEA), which binds CB_1_ with high affinity, but it is unable to activate CB_2_, and 2-arachydonoyl glycerol (2-AG), which acts as full agonist for both CBs [31,32]. It has been proven that AEA inhibits cell proliferation either in androgen-sensitive or insensitive prostate cancer cell lines via CB_1_ [9,10,33]. Furthermore, both AEA and 2-AG have been shown able to induce apoptosis both in tumor cell lines and in primary cultures of prostate cancer cells [7]. However, the contribution of an autocrine loop of endocannabinoids in modulating cancer aggressiveness it has been described only in a very few studies. Nithipatikom et al. showed that LNCaP, PC-3, and DU-145 produce 2-AG at high concentrations. In addition, these authors reported that the increase of endogenous 2-AG, induced by blocking its metabolism, inhibits androgen-insensitive prostate cancer cells invasion, while LNCaP invasiveness is unaffected [33]. Accordingly, we decided to investigate how the blocking of CB_1_ and CB_2_ with AM281 and JTE-907 could impair PC-3, and DU-145 cells migration. Tumor cells were treated with different concentrations of CBs antagonists, administrated alone or in combination. As a control, we used cells maintained in serum-deprived medium (Untr). Our results show that in androgen-insensitive cell lines, a lower dose of AM281 (0.5 µM) significantly reduces tumor cell migration, while the concentration of 1 µM restores the number of migrating cells at the same level of the control (Untr), in both PC-3 and DU-145 cells (Figure 5a,b). On the contrary, CB_2_ antagonist JTE-907 does not elicit different response at the different tested doses, showing a better inhibitory outcome at 1 µM. The response to the treatment in terms of cancer cell survival has been also shown to exclude toxic effects (Figure S1d). Based on these results, we therefore evaluated if CAFs’ reactivity could also be regulated by an autocrine self-sustaining loop of endocannabinoids. Our results show that CB_2_ impairment by JTE 1 µM (administrated alone or in combination with AM281), strongly reduces α-SMA expression (Figure 5c), suggesting a role of the endocannabinoids-mediated signalling in maintaining the CAFs active phenotype. 

## 3. Discussion

To date, an increasing number of preclinical studies have reported exciting anticancer properties of cannabinoid-related drugs, both in vitro and in xenograft models of several human cancers, including prostate cancer [1,2,3,4,5,6,7,8,9,10,11,12,13]. Interestingly, cannabinoids have been shown to selectively target tumor cells, which upregulate CBs compared with their healthy counterparts. Furthermore, in non-transformed cells that constitutively express CB_1_ and CB_2_, cannabinoids modulate cell-survival and cell-death pathways differently than in tumor cells. The best-established example is that of glioma cells and astrocytes. In glioma cells, CB_1_ activation induces de novo ceramide synthesis, which correlates with inhibition of Akt survival pathway and cell death [34,35,36]. On the contrary, in astrocytes CB_1_-mediated pathway is associated with Akt phosphorylation and cell survival [37,38]. This differential response has been reported also in rat thyroid epithelial cell lines and skin carcinoma cells [39,40]. Our data now reinforce this outcome selectivity of cannabinoids, showing that WIN 55-212.2 mesylate at concentrations higher than 5 µM induces cell death in prostate cancer cell lines, both androgen-sensitive and insensitive, without affecting healthy prostate epithelial cells (Figure 1a). Similarly, CBD 5 µM triggers cell death in cancer cell lines, while PNT-1 viability is not affected. It is not surprising that in response to cannabinoids’ concentration being higher than 30 µM, cell survival tends to increase in both healthy and cancer cells. Indeed, several studies have demonstrated that doses and the duration of the treatment are determinant factors to induce anti-neoplastic or pro-tumoral activity, which also depends on the agonist administered and the type of tumor [41,42,43,44]. This effect might rely, at least in part, on cannabinoids’ capacity to form homo- or hetero-oligomers in response to various agonists, interfering with the activation of other G signaling proteins different from inhibitory G proteins normally coupled to CB_1_ and CB_2_ [3,45]. However, the molecular basis of this “yin and yang” behavior remain an open question in cannabinoid field.

Since TME is now recognized as a hallmark of cancer, and stromal cells coevolve with tumor cells and sustain tumor progression [46], assessing the effect of cannabinoids on the microenvironmental components is of exciting interest. Here, we showed for the first time that patient-derived CAFs upregulate CBs compared to HPFs isolated from the healthy region (Figure 2a,b). This result is consistent with the increased expression of CB_1_ and CB_2_ in HPFs in response to tumor-secreted inflammatory cytokines (Figure 2c–e). In particular, HPFs and CAFs exhibit a different expression profile of CBs: CB_2_ expression is more than 2-fold higher in CAFs than in HPFs, CB_1_ is upregulated in CAFs, although to lesser extent, and TRPV1 is weakly expressed in both cell types. However, the treatment with WIN 55-212.2 mesylate does not affect cell survival, while CBD strongly impairs fibroblast viability (Figure 3a). These results suggest that CBD-mediated cell death could be dependent on signalling from non-canonical CBs, such as PPAR-γ. In keeping with that theme, Ramer et al. recently demonstrated that CBD induces apoptosis in primary cultures and cell lines of lung carcinoma via cyclooxygenase 2 and PPAR-γ [47]. Fibroblasts’ response to WIN 55-212.2 mesylate is supported by several studies reporting that the effect of THC and its analogues on cell survival is mediated by CB_1_, while CB_2_ regulates its anti-inflammatory properties [9,48,49,50,51]. We proved that treatment with WIN 55-212.2 mesylate strongly affects the CAFs’ phenotype and their ability to increase tumor cell invasiveness, via the CB_2_ receptor (Figure 3b–g and Figure 4). The controversial effect of CBD on activated stromal cells invasion should be further investigated to clarify its role on the modulation of stromal reactivity and the expression of other CBD sensitive receptors, and the role of CBD as an inverse agonist/antagonist should be evaluated. Our results are supported by previous studies demonstrating that cannabinoids are able to reduce inflammation and modulate wound healing processes in mouse models of autoimmune disorders [52]. If we consider cancer as a state of chronic inflammation, our data strongly reinforce the use of cannabinoids as a next generation treatment for cancer.

Finally, it has been widely demonstrated that alterations in endocannabinoid system correlate with tumor onset and progression [1,2,21,22,53]. Then, we wondered whether in addition to the inhibitory effect triggered by targeting CB_1_ and CB_2_ with synthetic agonists, the blocking of endogenous system could affect cancer and stromal reactivity. Our results suggest that both migration abilities of androgen-insensitive prostate cancer cells and prostate CAFs reactivity could be in part sustained by an autocrine self-sustaining loop of endocannabinoids (Figure 5). Interestingly, the involvement of CB_2_ has been demonstrated in tumor and stromal cells, as observed by the decrease of both cancer cell migration and CAFs α-SMA expression following the treatment with the selective antagonist JTE-907. In contrast, the treatment with AM281, the selective antagonist of CB_1_, showed, in tumor cells, opposite effects at 0.5 and 1 µM, suggesting that the biological effects of CB_1_ impairment could be dependent on the concentration and cell type, as already reported in the literature. Indeed, Endsley et al. showed that 2-AG may exert anti- and pro-invasive properties on prostate cancer cells depending on concentration [10,12]. They showed that autocrine 2-AG exhibited an anti-invasive effect in androgen-independent prostate cancer cells [10]. In contrast, when 2-AG was exogenously added at 1 µM, PC-3 and DU-145 cells invasiveness significantly increased [12]. Accordingly, both endogenous CB_1_ and CB_2_ agonists and antagonists may exert different effects depending on the concentration. Further studies will be needed to clarify this response. 

Overall, our results stress the importance of the endocannabinoid system in prostate cancer progression and reinforce the therapeutic potential of WIN 55-212.2 mesylate in prostate cancer treatment, since it is able to simultaneously impact on cancer cells and stromal compartments. Further studies will be needed to clarify the role of endocannabinoids in tumor progression and aggressiveness in order to develop new possible therapeutic approaches to counteract tumor progression.

## 4. Materials and Methods 

### 4.1. Antibodies and Reagents

For the western blot analysis, the following antibodies were used: anti- α -SMA (#number A5228) from Sigma-Aldrich (Darmstadt, Germany); anti-CB_1_ (#number ACR-001) and anti-TRPV1 (#number ACC-030) from Alomone Labs (Jerusalem BioPark (JBP), Jerusalem, Israel); anti-CB_2_ (#number sc-293188), anti-GAPDH (#number sc-365062), and secondary antibodies from Santa Cruz Biotechnology (Dallas, TX, USA); anti-ERK1/2 (#number 9102), pERK1/2 (#number 9101), SMAD2/3 (#number D7G7) and pSMAD2/3 (#number D27F4) from Cell signaling (Danvers, MA, USA). All primary antibodies, except for anti-CB_1_ (1:500), were used at 1:1000. CBD (#number 90899) was purchased from Sigma-Aldrich (Darmstadt, Germany), and WIN 55-212.2 mesylate (#1038) was from Tocris Bioscience (prior Ministerial Decree SP/096, released on September 11^th^ 2018) (Bristol, UK). Recombinant TGF-β 1 (#number 130-095-067) was provided from Miltenyi Biotec (Bergisch Gladbach, Germany), human TNF-α (#number 300-01A) and IL-6 (#number GMP200-06) were from PeproTech (London, UK). TGF- β receptor inhibitor (A8301, #number 2939), and CB_1_ and CB_2_ antagonists (AM281, #number 1115 and JTE-907, #number 2479, respectively), were from Tocris Bioscience (Bristol, UK). Blocking antibodies for IL-6 (α-IL-6, #number mabg-hil6-3) were from InvivoGen (San Diego, CA, USA). Matrigel™, Basement Membrane Matrix (#number 356234) was from BD Biosciences (Allschwil, Swiss).

### 4.2. Cell Models and Cell Cultures

LNCaP and PNT-1 cells were purchased from Sigma-Aldrich (Darmstadt, Germany); PC-3 and DU-145 cells were from the American Type Culture Collection (ATCC, Manassas, VA, USA). PC-3 cells were grown in HAM’S F12 medium; DU-145 cells were cultured in DMEM, LNCaP and PNT-1 cells were cultured in RPMI 1640. Human prostate fibroblasts were isolated from aggressive prostate cancer-bearing patients (Gleason score 4+5, 4+4, grade ≤ pT3), with HPFs and CAFs deriving from the healthy region and the intra-tumoral area, respectively. The isolated HPFs and CAFs were maintained in DMEM. The patients enrolled in the study underwent prostatectomy without receiving prior hormone deprivation therapy. All the enrolled patients provided their signed informed consent, in agreement with the Ethics Committee of the Azienda Ospedaliera Universitaria Careggi (approval code: CEAVC – 2018-256, on October 10th 2018). All the cells used were grown at 37 °C/5% CO_2_. All cultured media were purchased from EuroClone (Milano, Italy) and supplemented with 10% fetal bovin serum (FBS, EuroClone, Milano, Italy), 1% penicillin/streptomycin and 2 mM glutamine (Sigma-Aldrich, Darmstadt, Germany). 

### 4.3. Conditioned Media Preparation

Conditioned media were obtained from sub-confluent cells maintained in serum-free medium (St Med) for 48 h. Once collected, conditioned media were filtered and used freshly or frozen at −80 °C until use.

### 4.4. Western Blot Analysis

Cells were lysed in Ripa buffer (Thermo Fisher, Waltham, MA, USA), after the addition of protease and phosphatase inhibitor cocktails (#number P1860 and #number 026M4058V, Sigma-Aldrich, Darmstadt, Germany), and total proteins were quantified with BCA assay (#number SLCC1765, Sigma-Aldrich, Darmstadt, Germany). Between 10 and20 µg of total protein lysate was loaded on each precast gel (4–20% acrylamide, mini-PROTEAN TGX Stain-Free, Bio-Rad, Hercules, CA, USA), and that was transferred onto a membrane by the Trans-Blot Turbo Transfer System (Bio-Rad, Hercules, CA, USA). The immunoblot was performed as previously described [14] and was analyzed by Amersham Imager 600 (GE, Healthcare Life Sciences, Marlborough, MA, USA).

### 4.5. Crystal Violet Staining

HPFs and CAFs (3 × 10^4^ cells) or prostate cancer cells (1 × 10^4^ cells) were seeded in 24-multiwell plates and maintained in St Med for 24 h, before receiving WIN 55-212.2 mesylate or CBD, at concentrations ranging from 0.5 to 100 µM. After 24 h, cells were washed twice with PBS, fixed with formaldehyde 4% (*v*/*v*), and then incubated at room temperature with crystal violet solution (crystal violet 0.5% (*w*/*v*) and methanol 20% (*v*/*v*)). After the incubation, the cells were washed with PBS and treated with SDS 2% (*w*/*v*) for 1 h at 37 °C. The absorbance at 595 nm was measured.

### 4.6. Gelatin Zymography

Conditioned media from CAFs and HPFs activated in vitro by TGF- β 10 ng/mL, treated or not for 24 h with cannabinoids (WIN 55-212.2 mesylate and CBD 2.5 µM) and CBs antagonists (AM281 and JTE-907 0.5 µM), were collected, 10-fold concentrated with Amicon centrifugal filter (UFC800324, Merck-Millipore, Burlington, MA, USA) and analyzed by gelatin zymography as previously described [54].

### 4.7. Boyden Chamber Assay

Invasion and migration assays were performed in transwells (8 µm pore polyvinylpyrrolidone-free polycarbonate filters, #number CC3422, Corning, Corning, NY, USA) with or without pre-coating with 50 µg/cm^2^ of reconstituted Matrigel, as previously described [27]. Briefly, CAFs and HPFs activated in vitro by TGF- β 10 ng/mL, were serum-starved for 24 h and then treated with WIN 55-212.2 mesylate and CBD 2.5 µM for an additional 24 h. Then, 1 × 10^5^ fibroblasts were added to the upper chamber of Matrigel-coated transwells and allowed to invade at 37 °C for 24 h towards complete medium (20% FBS). 

PC-3 cells were incubated at 37 °C for 48 h with conditioned media from CAFs treated or not with WIN 55-21.2 mesylate and CBD 2.5 µM. Then, 3 × 10^4^ cells were added to the upper chamber of Matrigel-coated transwells and allowed to invade at 37 °C for 16 h towards complete medium (10% FBS).

To investigate the effect of an endocannabinoids autocrine self-sustaining loop on cell migration, PC-3 or DU-145 cells were treated at 37 °C for 24 h with AM281 and JTE-907 at 0.5 µM and 1 µM. The pharmacological agents were administrated alone or in combination and added again during the migration assay. 3 × 10^4^ PC-3 and DU-145 cells were allowed to migrate in transwells at 37 °C for 16 h towards complete medium (10% FBS). 

The migration/invasion was reported as fold change (FC) compared with the starved control (Untr).

### 4.8. Statistical Analysis

Statistics were performed using Prism 8 (GraphPad Software, San Diego, CA, USA). Data were represented as the means ± SEM of at least three independent experiments. Statistical analysis was performed by Student’s *t* test or one-way analysis of variance (ANOVA) followed by post-hoc test. The significance was set at *p* value < 0.05.

## Supplementary Materials

Supplementary materials can be found at https://www.mdpi.com/1422-0067/21/3/787/s1.

## Abbreviations

α-SMA	Alpha-smooth muscle actin
2-AG	2-arachydonoyl glycerol
5-HT	5-hydroxytryptamine
AEA	Anandamide
CAFs	Cancer-associated fibroblasts
CB1	Cannabinoid receptor 1
CB2	Cannabinoid receptor 2
CBD	Cannabidiol
EMT	Epithelial to mesenchymal transition
HPFs	Healthy prostate fibroblasts
IL-6	Interleukin-6
MMP-2	Matrix metalloproteinase-2
PPAR-γ	Peroxisome proliferator-activated receptor gamma
TGF-β	Transforming growth factor beta
THC	Delta-9-tetrahydrocannabinol
TME	Tumor microenvironment
TNF-α	Tumor necrosis factor alpha
TRPV1	Vanilloid receptor 1

## References

[1] Hermanson D.J., Marnett L.J. (2011). Cannabinoids, endocannabinoids, and cancer. Cancer Metastasis Rev..

[2] Guzmán M. (2003). Cannabinoids: Potential anticancer agents. Nat. Rev. Cancer.

[3] Velasco G., Sánchez C., Guzmán M. (2016). Anticancer mechanisms of cannabinoids. Curr. Oncol..

[4] Ramer R., Hinz B. (2016). Antitumorigenic targets of cannabinoids—Current status and implications. Expert Opin. Ther. Targets.

[5] Fraguas-Sánchez A., Fernández-Carballido A., Torres-Suárez A. (2016). Phyto-, endo- and synthetic cannabinoids: Promising chemotherapeutic agents in the treatment of breast and prostate carcinomas. Expert Opin. Investig. Drugs.

[6] Sarfaraz S., Afaq F., Adhami V.M., Mukhtar H. (2005). Cannabinoid Receptor as a Novel Target for the Treatment of Prostate Cancer. Cancer Res..

[7] Orellana-Serradell O., Poblete C.E., Sanchez C., Castellón E.A., Gallegos I., Huidobro C., Llanos M.N., Contreras H.R. (2015). Proapoptotic effect of endocannabinoids in prostate cancer cells. Oncol. Rep..

[8] Melck D., de Petrocellis L., Orlando P., Bisogno T., Laezza C., Bifulco M., Di Marzo V. (2000). Suppression of nerve growth factor Trk receptors and prolactin receptors by endocannabinoids leads to inhibition of human breast and prostate cancer cell proliferation. Endocrinology.

[9] Mimeault M., Pommery N., Wattez N., Bailly C., Hénichart J.P. (2003). Anti-proliferative and apoptotic effects of anandamide in human prostatic cancer cell lines: Implication of epidermal growth factor receptor down-regulation and ceramide production. Prostate.

[10] Nithipatikom K., Endsley M.P., Isbell M.A., Falck J.R., Iwamoto Y., Hillard C.J., Campbell W.B. (2004). 2-arachidonoylglycerol: A novel inhibitor of androgen-independent prostate cancer cell invasion. Cancer Res..

[11] Sreevalsan S., Joseph S., Jutooru I., Chadalapaka G., Safe S.H. (2011). Induction of apoptosis by cannabinoids in prostate and colon cancer cells is phosphatase dependent. Anticancer Res..

[12] Endsley M.P., Aggarwal N., Isbell M.A., Wheelock C.E., Hammock B.D., Falck J.R., Campbell W.B., Nithipatikom K. (2007). Diverse roles of 2-arachidonoylglycerol in invasion of prostate carcinoma cells: Location, hydrolysis and 12-lipoxygenase metabolism. Int. J. Cancer.

[13] Roberto D., Klotz L.H., Venkateswaran V. (2019). Cannabinoid WIN 55,212-2 induces cell cycle arrest and apoptosis, and inhibits proliferation, migration, invasion, and tumor growth in prostate cancer in a cannabinoid-receptor 2 dependent manner. Prostate.

[14] Giannoni E., Bianchini F., Masieri L., Serni S., Torre E., Calorini L., Chiarugi P. (2010). Reciprocal activation of prostate cancer cells and cancer-associated fibroblasts stimulates epithelial-mesenchymal transition and cancer stemness. Cancer Res..

[15] Fiaschi T., Marini A., Giannoni E., Taddei M.L., Gandellini P., De Donatis A., Lanciotti M., Serni S., Cirri P., Chiarugi P. (2012). Reciprocal metabolic reprogramming through lactate shuttle coordinately influences tumor-stroma interplay. Cancer Res..

[16] Giannoni E., Taddei M.L., Parri M., Bianchini F., Santosuosso M., Grifantini R., Fibbi G., Mazzanti B., Calorini L., Chiarugi P. (2013). EphA2-mediated mesenchymal-amoeboid transition induced by endothelial progenitor cells enhances metastatic spread due to cancer-associated fibroblasts. J. Mol. Med..

[17] Comito G., Giannoni E., Segura C.P., Barcellos-de-Souza P., Raspollini M.R., Baroni G., Lanciotti M., Serni S., Chiarugi P. (2014). Cancer-associated fibroblasts and M2-polarized macrophages synergize during prostate carcinoma progression. Oncogene.

[18] Giannoni E., Taddei M.L., Morandi A., Comito G., Calvani M., Bianchini F., Richichi B., Raugei G., Wong N., Tang D. (2015). Targeting stromal-induced pyruvate kinase M2 nuclear translocation impairs oxphos and prostate cancer metastatic spread. Oncotarget.

[19] Ippolito L., Morandi A., Taddei M.L., Parri M., Comito G., Iscaro A., Raspollini M.R., Magherini F., Rapizzi E., Masquelier J. (2019). Cancer-associated fibroblasts promote prostate cancer malignancy via metabolic rewiring and mitochondrial transfer. Oncogene.

[20] Comito G., Iscaro A., Bacci M., Morandi A., Ippolito L., Parri M., Montagnani I., Raspollini M.R., Serni S., Simeoni L. (2019). Lactate modulates CD4+ T-cell polarization and induces an immunosuppressive environment, which sustains prostate carcinoma progression via TLR8/miR21 axis. Oncogene.

[21] Bifulco M., Di Marzo V. (2002). Targeting the endocannabinoid system in cancer therapy: A call for further research. Nat. Med..

[22] Ramer R., Schwarz R., Hinz B. (2019). Modulation of the Endocannabinoid System as a Potential Anticancer Strategy. Front. Pharmacol..

[23] Tuxhorn J.A., Ayala G.E., Smith M.J., Smith V.C., Dang T.D., Rowley D.R. (2002). Reactive stroma in human prostate cancer: Induction of myofibroblast phenotype and extracellular matrix remodeling. Clin. Cancer Res..

[24] Barron D.A., Rowley D.R. (2012). The reactive stroma microenvironment and prostate cancer progression. Endocr. Relat. Cancer.

[25] Doldi V., Callari M., Giannoni E., D’Aiuto F., Maffezzini M., Valdagni R., Chiarugi P., Gandellini P., Zaffaroni N. (2015). Integrated gene and miRNA expression analysis of prostate cancer associated fibroblasts supports a prominent role for interleukin-6 in fibroblast activation. Oncotarget.

[26] Jean-Gilles L., Braitch M., Latif M.L., Aram J., Fahey A.J., Edwards L.J., Robins R.A., Tanasescu R., Tighe P.J., Gran B. (2015). Effects of pro-inflammatory cytokines on cannabinoid CB1 and CB2 receptors in immune cells. ACTA Physiol..

[27] Pietrovito L., Leo A., Gori V., Lulli M., Parri M., Becherucci V., Piccini L., Bambi F., Taddei M.L., Chiarugi P. (2018). Bone marrow-derived mesenchymal stem cells promote invasiveness and transendothelial migration of osteosarcoma cells via a mesenchymal to amoeboid transition. Mol. Oncol..

[28] Peres F.F., Lima A.C., Hallak J.E.C., Crippa J.A., Silva R.H., Abílio V.C. (2018). Cannabidiol as promising strategy to treat and prevent movement disorders?. Front. Pharmacol..

[29] Giannoni E., Bianchini F., Calorini L., Chiarugi P. (2011). Cancer associated fibroblasts exploit reactive oxygen species through a proinflammatory signature leading to epithelial mesenchymal transition and stemness. Antioxid. Redox. Signal..

[30] Pistore C., Giannoni E., Colangelo T., Rizzo F., Magnani E., Muccillo L., Giurato G., Mancini M., Rizzo S., Riccardi M. (2017). DNA methylation variations are required for epithelial-to-mesenchymal transition induced by cancer-associated fibroblasts in prostate cancer cells. Oncogene.

[31] Schwarz R., Ramer R., Hinz B. (2018). Targeting the endocannabinoid system as a potential anticancer approach. Drug Metab. Rev..

[32] Maccarrone M., Bab I., Bíró T., Cabral G.A., Dey S.K., Di Marzo V., Konje J.C., Kunos G., Mechoulam R., Pacher P. (2015). Endocannabinoid signaling at the periphery: 50 years after THC. Trends Pharmacol. Sci..

[33] Nithipatikom K., Isbell M.A., Endsley M.P., Woodliffm J.E., Campbell W.B. (2011). Anti-proliferative effect of a putative endocannabinoid, 2-arachidonylglyceryl ether in prostate carcinoma cells. Prost. Other. Lipid Mediat..

[34] Carracedo A., Lorente M., Egia A., Blázquez C., García S., Giroux V., Malicet C., Villuendas R., Gironella M., González-Feria L. (2006). The stress-regulated protein p8 mediates cannabinoid-induced apoptosis of tumor cells. Cancer Cell.

[35] Galve-Roperh I., Sánchez C., Cortés M.L., Gómez del Pulgar T., Izquierdo M., Guzmán M. (2000). Anti-tumoral action of cannabinoids: Involvement of sustained ceramide accumulation and extracellular signal-regulated kinase activation. Nat. Med..

[36] Ellert-Miklaszewska A., Ciechomska I., Kaminska B. (2013). Cannabinoid signaling in glioma cells. Adv. Exp. Med. Biol..

[37] Galve-Roperh I., Aguado T., Palazuelos J., Guzmán M. (2008). Mechanisms of control of neuron survival by the endocannabinoid system. Curr. Pharm. Des..

[38] Salazar M., Carracedo A., Salanueva I.J., Hernández-Tiedra S., Lorente M., Egia A., Vázquez P., Blázquez C., Torres S., García S. (2009). Cannabinoid action induces autophagy-mediated cell death through stimulation of ER stress in human glioma cells. J. Clin. Invest..

[39] Bifulco M., Laezza C., Portella G., Vitale M., Orlando P., De Petrocellis L., Di Marzo V. (2001). Control by the endogenous cannabinoid system of ras oncogene-dependent tumor growth. FASEB J..

[40] Casanova M.L., Blázquez C., Martínez-Palacio J., Villanueva C., Fernández-Aceñero M.J., Huffman J.W., Jorcano J.L., Guzmán M. (2003). Inhibition of skin tumor growth and angiogenesis in vivo by activation of cannabinoid receptors. J. Clin. Invest..

[41] De Petrocellis L., Melck D., Palmisano A., Bisogno T., Laezza C., Bifulco M., Di Marzo V. (1998). The endogenous cannabinoid anandamide inhibits human breast cancer cell proliferation. Proc. Natl. Acad. Sci. USA.

[42] McKallip R.J., Nagarkatti M., Nagarkatti P.S. (2005). Delta-9-tetrahydrocannabinol enhances breast cancer growth and metastasis by suppression of the antitumor immune response. J. Immunol..

[43] Sánchez M.G., Sánchez A.M., Ruiz-Llorente L., Díaz-Laviada I. (2003). Enhancement of androgen receptor expression induced by (R)-methanandamide in prostate LNCaP cells. FEBS Lett..

[44] Hart S., Fischer O.M., Ullrich A. (2004). Cannabinoids induce cancer cell proliferation via tumor necrosis factor alpha-converting enzyme (TACE/ADAM17)-mediated transactivation of the epidermal growth factor receptor. Cancer Res..

[45] Bifulco M., Laezza C., Pisanti S., Gazzerro P. (2006). Cannabinoids and cancer: Pros and cons of an antitumour strategy. Br. J. Pharmacol..

[46] Chiarugi P. (2013). Cancer-associated fibroblasts and macrophages: Friendly conspirators for malignancy. Oncoimmunology.

[47] Ramer R., Heinemann K., Merkord J., Rohde H., Salamon A., Linnebacher M., Hinz B. (2013). COX-2 and PPAR-γ confer cannabidiol-induced apoptosis of human lung cancer cells. Mol. Cancer Ther..

[48] Silva A.G., Lopes C.F.B., Carvalho Júnior C.G., Thomé R.G., Dos Santos H.B., Reis R., Ribeiro R.I.M.A. (2019). WIN55,212-2 induces caspase-independent apoptosis on human glioblastoma cells by regulating HSP70, p53 and Cathepsin, D. Toxicol. Vitro.

[49] Hiebel C., Kromm T., Stark M., Behl C. (2014). Cannabinoid receptor 1 modulates the autophagic flux independent of mTOR- and BECLIN1-complex. J. Neurochem..

[50] Emery S.M., Alotaibi M.R., Tao Q., Selley D.E., Lichtman A.H., Gewirtz D.A. (2014). Combined antiproliferative effects of the aminoalkylindole WIN55,212-2 and radiation in breast cancer cells. J. Pharmacol. Exp. Ther..

[51] Caffarel M.M., Andradas C., Mira E., Pérez-Gómez E., Cerutti C., Moreno-Bueno G., Flores J.M., García-Real I., Palacios J., Mañes S. (2010). Cannabinoids reduce ErbB2-driven breast cancer progression through Akt inhibition. Mol. Cancer.

[52] Katchan V., David P., Shoenfeld Y. (2016). Cannabinoids and autoimmune diseases: A systematic review. Autoimmun. Rev..

[53] Pyszniak M., Tabarkiewicz J., Łuszczki J.J. (2016). Endocannabinoid system as a regulator of tumor cell malignancy—Biological pathways and clinical significance. Onco. Targets Ther..

[54] Taddei M.L., Giannoni E., Morandi A., Ippolito L., Ramazzotti M., Callari M., Gandellini P., Chiarugi P. (2014). Mesenchymal to amoeboid transition is associated with stem-like features of melanoma cells. Cell Commun. Signal..

