# Supplementary figures and images for: Treatment with Cannabinoids as a Promising Approach for Impairing Fibroblast Activation and Prostate Cancer Progression
Source: Int J Mol Sci. 2020 Jan 25;21(3):787. doi: 10.3390/ijms21030787 (PMC7037293; doi:10.3390/ijms21030787)

Figure S1

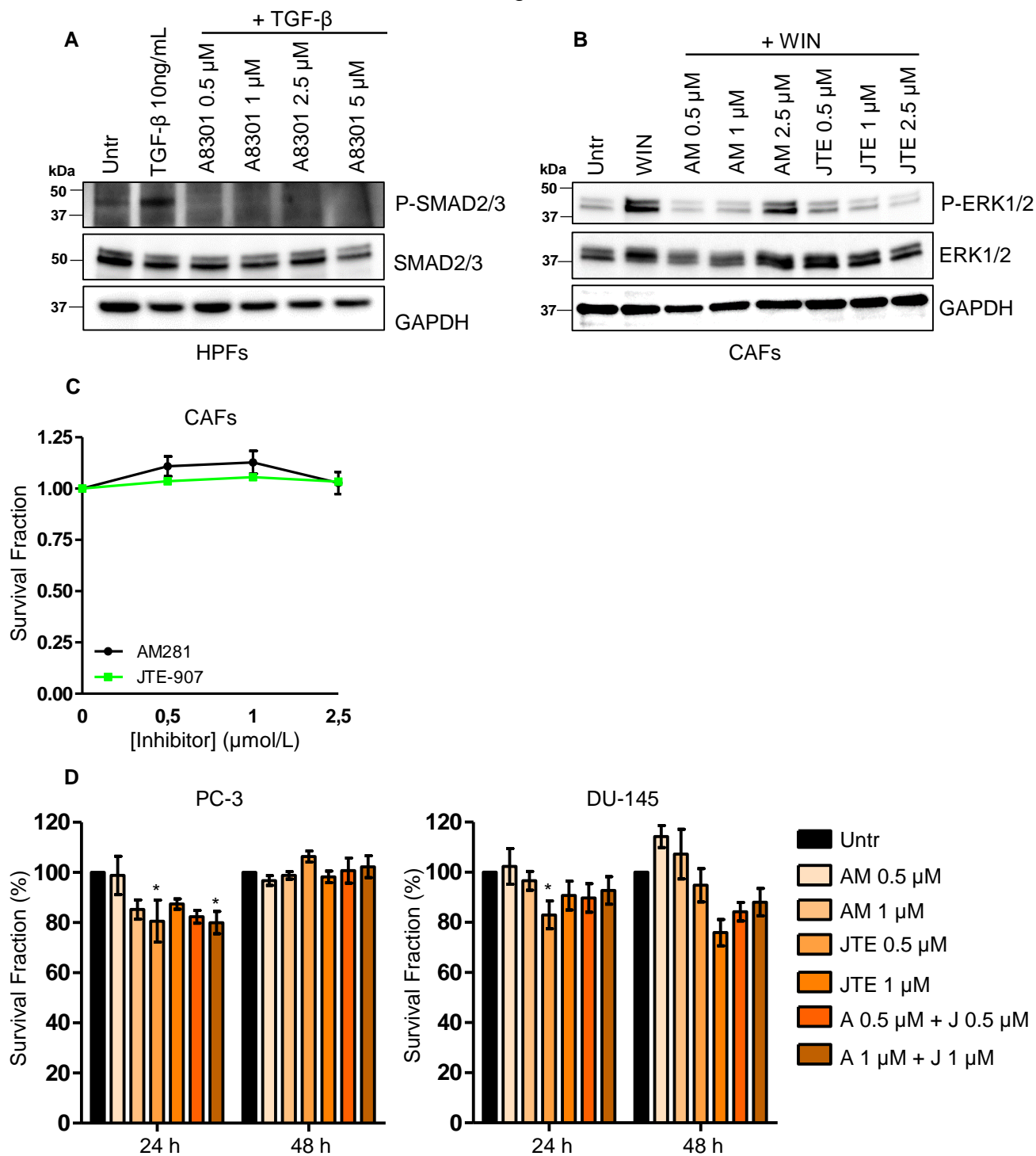

Supplement: Supplementary file 1 [file ijms-21-00787-s001.pdf]
